# The determinant factors for macroinvertebrate assemblages in a recreational river in Negeri Sembilan, Malaysia

**DOI:** 10.1007/s10661-021-09196-7

**Published:** 2021-06-08

**Authors:** Mohd Noorazhan Azis, Azlan Abas

**Affiliations:** Centre for Research and Development, Social and Environment (SEEDS), Faculty of Social Sciences and Humanities, Universiti Kebangsaan Malaysia, Bangi, Malaysia

**Keywords:** Ecological indicator, Environmental factor, Environmental management, Water quality, River ecosystem

## Abstract

The determinant factors for macroinvertebrate assemblages in river ecosystems are varied and are unique and specific to the type of macroinvertebrate family. This study aims to assess the determinant factors for macroinvertebrate assemblages in a recreational river. The study was conducted on the Ulu Bendul River, Negeri Sembilan, Malaysia. A total of ten sampling stations were selected. The research methodology included (1) water quality measurement, (2) habitat characterization, and (3) macroinvertebrate identification and distribution analysis. The statistical analysis used in this study was canonical correspondence analysis (CCA) to represent the relationship between the environmental factors and macroinvertebrate assemblages in the recreational river. This study found that most of the families of macroinvertebrates were very dependent on the temperature, DO, NH_3_-N, type of riverbed, etc. All of these factors are important for the survival of the particular type of macroinvertebrate, plus they are also important for selecting egg-laying areas and providing suitable conditions for the larvae to grow. This study advises that improved landscape design for watershed management be implemented in order to enhance water quality and physical habitats, and hence the protection and recovery of the macroinvertebrate biodiversity.

## Introduction

Environmental factors such as temperature, pH, oxygen concentration, soil, and rock structure are always important considerations for the richness and distribution of living things in an ecosystem (Abas et al., 2020; Leps et al., 2015). Different types of organisms require specific environmental factors for their survival. Changes in the environmental factors of an ecosystem may introduce some new communities and also cause the loss of others. But it all depends on the organism, where some organisms may be really sensitive toward the environmental changes and some may be resistant to the change and can stay and live with the new environmental conditions. According to Lin et al. (2016), only a few organism communities can exist in various environmental conditions and also have different distribution and abundance of its species. This kind of organism or living thing is well-fitted for the role of biological indicator, for example the macroinvertebrate community assemblage as a biological indicator for water quality (Kasihmuddin & Cob, 2021; Wang & Tan, 2017).

Macroinvertebrates have been widely employed as indicators of stream conditions due to their vulnerability to disturbance gradients (e.g., emissions, habitat destruction, and toxicants) (Ahmad et al., 2015; Mereta et al., 2020). Macroinvertebrates are exothermic (or cold-blooded) and may be aquatic or terrestrial, the aquatic organisms often being larval or nymphal forms of otherwise terrestrial species. Their physical appearance varies widely, with some, like crayfish, having an exoskeleton and others, like snails, having a shell. Still others, such as leeches, have soft flesh with no support or protective structure (Clarke et al., 2008). Macroinvertebrates are found in almost every freshwater environment on the planet, including some that appear to be inactive. They serve as a substantial source of food for other species such as amphibians, birds, reptiles, and fish at the bottom of the aquatic food chain (O’Brien et al., 2016). Because macroinvertebrates are nonmigratory and spend their whole lives in a confined region, they frequently exhibit the consequences of habitat change. As a result, they are excellent markers of environmental health, particularly in streams and other waterways (Ahmad et al., 2015; Li et al., 2018; Wang & Tan, 2017). They can also be sampled and identified using inexpensive technology, making them ideal for environmental biomonitoring. Waterway health may be determined in general based on the kind and number of macroinvertebrate families present. Certain macroinvertebrate groups are more susceptible to pollution than others; the presence of numerous such species often signals clean water (Shabani et al., 2019). For example, the larvae of spiny crawler mayflies (family Ephemerellidae) have a tolerance value of 1 and can dwell only in the most pristine environments. Leeches, by comparison, are equipped with a high tolerance value of 10 and therefore are more tolerant of pollution (Fierro et al., 2018; Johnson et al., 2013; Ntislidou et al., 2018).

The presence of water is critical not only for the ecological environment but also for economic development as a tourism resource, such as for numerous recreational activities (e.g., fishing, water rafting, swimming, etc.) (Jang, 2016). Recreational activities on rivers are varied, depending on the type of the river itself. Through various recreational activities, rivers can become major contributors to local economic development and also to changes in environmental conditions (Kistemann et al., 2016). Recreational activity in river areas will usually affect the environmental conditions through several aspects such as soil erosion and compaction, damage to vegetation, disturbance to wildlife, water pollution, increased fire frequency, vandalism, and noise (Van Hoey et al., 2010). On the other hand, previous research (Faudzi et al., 2021) has shown that the degradation of environmental conditions of the river ecosystem will lead to the loss of its ecosystem services value, especially the cultural value. This will be a hindrance for visitors and foreign tourists to come to the area, which will eventually dampen the local economic activities (Barnett et al., 2018).

The use of the macroinvertebrate community as a biological indicator for water quality, especially for river ecosystems, has been explored by numerous studies (Ahmad et al., 2015; Jang, 2016; Lin et al., 2016; Wang & Tan, 2017; Li et al., 2018; Wan Abdul Ghani et al., 2018). However, the determinant factors for macroinvertebrate assemblages remain vague, although a few studies have made an attempt at identifying the factors related to the assemblages (Liu et al., 2016; Wu et al., 2020; Zhao et al., 2019). Therefore, the research objectives of this study were to (1) examine the characteristics of habitats in the recreational river, (2) analyze the water quality parameters in the recreational river, (3) identify the species diversity richness and distribution of macroinvertebrates in the recreational river, and (4) assess the determinant factors for the macroinvertebrate assemblages in the recreational river. This study hypothesized that the benthic macroinvertebrate assemblages in the recreational river varied depending on specific determinant factors.

## Materials and methods

### Study area

The research was carried out on a recreational river in Ulu Bendul, Negeri Sembilan, Malaysia. The research location is located 20 km from Kuala Pilah and has the coordinates 2°44′25.7064″ N 102°14′55.9392″ E. The Ulu Bendul recreational river is located in Peninsular Malaysia, on the southern side of the Titiwangsa mountain range, and on the bank of the Batang Terachi River in the district of Kuala Pilah, Negeri Sembilan. According to a research by Idris et al. (2013), the rainforest reserve is thought to be 130 million years old. Camping, jungle hiking, fishing, and swimming are some of the outdoor activities that may be done in the forest. Climbers can explore the hilly terrain around the Ulu Bendul Recreational Forest.

**Fig. 1:**
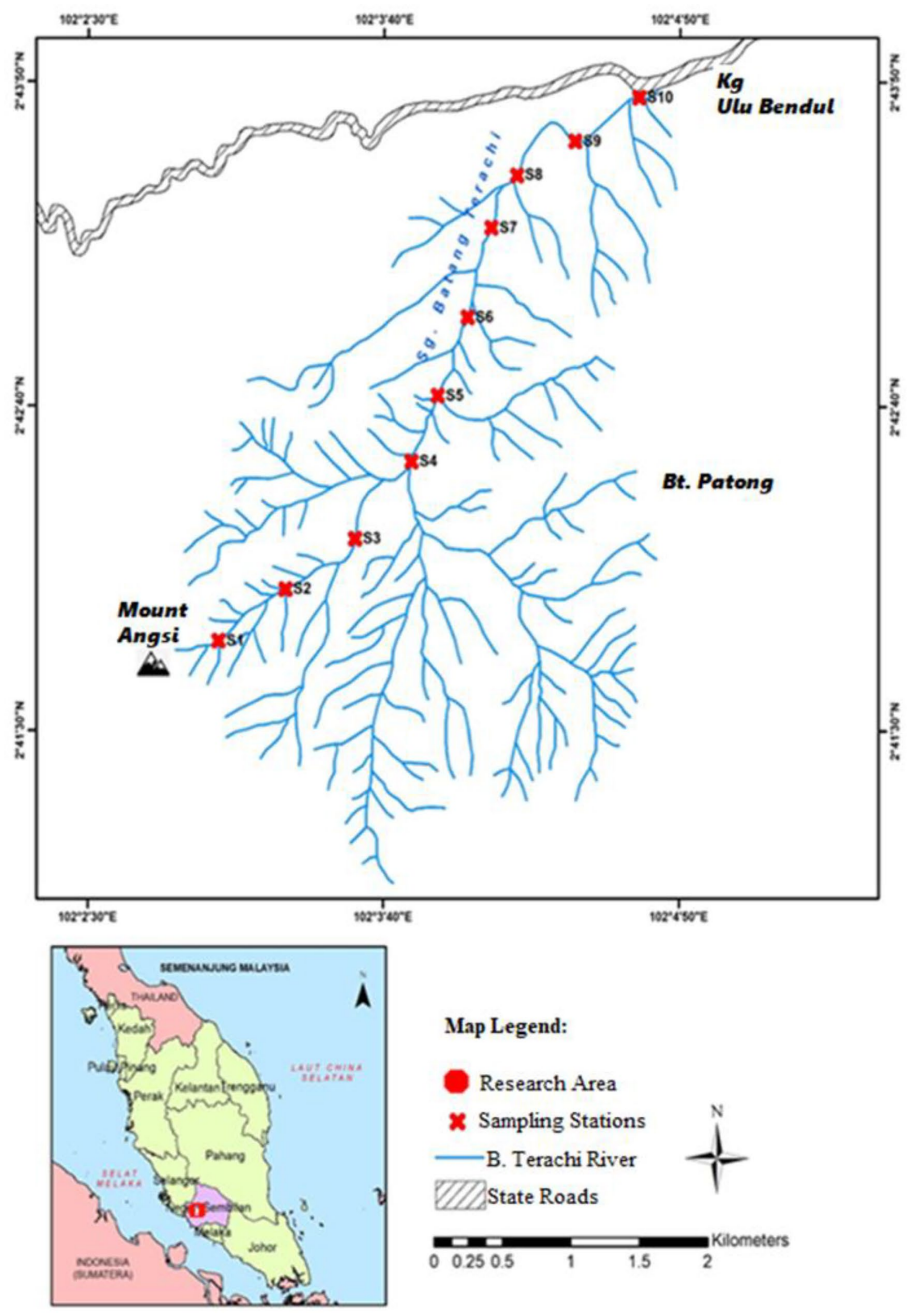
Map of sampling location

This study sampled ten sites on the tributaries of the Ulu Bendul recreational river (Fig. 1) on 18 March 2020. The study used a grab sampling design (100 m of distance between sampling station) to select all the sites from downstream to the upstream (Harmel et al., 2010).

### Sampling procedures

#### Habitat characterization

The habitat characterization was conducted by dividing the characteristics of the river into three main categories, which were (1) canopy cover on the river (both on the riverbank and the middle of the river), (2) riverbed (rock composition), and (3) physical features (temperature and depth) (Ahmad et al., 2015).

#### Water quality parameter analysis

Water samples were collected from each site prior to macroinvertebrate sampling, dark preserved in a portable refrigerator at 4 °C, and then taken to the laboratory for further analysis. A total of six parameters were measured in the river water samples; these were pH, dissolved oxygen (DO), total suspended solids (TSS), biochemical oxygen demand (BOD), chemical oxygen demand (COD), and ammoniacal nitrogen (NH_3_-N). All parameters were measured according to the Malaysia Water Quality Standard (Department of Environment, 2021). All analyses were performed in the laboratory following the standard methods for the analysis of water and wastewater (APHA, 1995), except for DO and pH which were analyzed using a water quality multiparameter instrument (YSI 556 MPS).

#### Macroinvertebrate sample identification

Macroinvertebrate samples were collected at three riffle and two pool habitats with Surber sampler nets (30 × 30 cm area and 250 μm mesh size) along a 100-m-long reach of the stream (Jørgensen et al., 2011; Wang et al., 2012). All five subsamples were pooled (giving a total sample area of 0.45 m^2^), field washed, and preserved in 4% formalin. This study also counted and identified all individuals to the lowest possible taxon, usually the genus level (Ahmad et al., 2015; Wang, 2002) in the laboratory.

This study then calculated the macroinvertebrate taxa richness, number of Ephemeroptera, Plecoptera, and Trichoptera (EPT) taxa, % EPT individuals, % intolerant individuals, and the Shannon–Wiener diversity index as biological response variables. These five biological metrics are the most widely used to represent macroinvertebrate richness, composition, tolerance, and diversity measurements that are sensitive to environmental change (Hawkins et al., 2010).

### Statistical analysis

This study used canonical correspondence analysis (CCA) using PAST software version 3.0. CCA is a multivariate method to elucidate the relationships between biological assemblages of species and their environment. CCA was used in this study to discover the relationships between the assemblages of macroinvertebrates, water quality parameters, and the habitat characterization (Faudzi et al., 2021).

## Results

### Habitat characterization

The habitat characterization was divided into three main features: (1) canopy cover (river bank and on top of the river), (2) riverbed (rock composition), and (3) physical features (temperature and depth). Based on Table 1, most of the sampling stations had 50% or more canopy cover at the riverbank. Meanwhile, on top of the river, the canopy cover at all of the sampling stations ranged between 0 and 40%. For the rock composition on the riverbed, all sampling stations recorded four types of rocks which were small rocks, large rocks, sand, and debris. The highest percentages were sand ranging between 40 and 80%, followed by large rocks (5–30%), small rocks (5–20%), and debris (5–20%). The highest temperature was recorded at sampling station no. 4 with 24.4 ºC, and the lowest temperature was at station no. 10 with 23.3 ºC. The greatest depth was recorded at station no. 8 with 0.45 m, and the shallowest was at station no. 5 with 0.17 m.

**Table 1:** Habitat characterization

Sampling station	Canopy cover		Riverbed				Physical	
	River bank (%)	Top of the river (%)	Small rocks (%)	Large rocks (%)	Sand (%)	Debris (%)	Depth (m)	Temperature (°C)
1	90	10	5	10	80	5	0.33	23.5
2	70	30	5	10	75	10	0.27	23.6
3	60	40	5	25	60	10	0.24	23.9
4	65	35	20	30	45	5	0.22	24.4
5	70	30	15	25	40	20	0.17	24.1
6	60	40	20	10	50	20	0.38	24.3
7	60	40	15	20	60	5	0.35	24.1
8	10	0	10	5	80	5	0.45	23.9
9	30	15	10	10	75	5	0.22	24
10	60	40	5	10	80	5	0.27	23.3
Average	58	28	11	15.5	64.5	9	0.3	24

### Water quality parameter measurement

This study measured six water quality parameters, which were pH, dissolved oxygen (DO), total suspended solid (TSS), biochemical oxygen demand (BOD), chemical oxygen demand (COD), and ammoniacal nitrogen (NH_3_-N). Based on Table 2, the DO concentration ranged between 1.93 and 5.24 mg/L, BOD ranged between 0.32 and 0.37 mg/L, COD ranged between 0 and 23 mg/L, NH_3_-N ranged between 0 and 0.18 mg/L, TSS ranged between 4 and 66 mg/L, and pH ranged between 6.45 and 7.40.

**Table 2:** Water quality measurement

Parameter	Sampling station										Average
	1	2	3	4	5	6	7	8	9	10	
DO (mg/l)	2.60	1.93	3.01	2.02	2.04	2.63	5.24	2.53	2.51	2.56	2.71
BOD (mg/l)	0.34	0.32	0.33	0.33	0.34	0.34	0.36	0.36	0.35	0.37	0.34
COD (mg/l)	18	4	22	16	7	23	0	13	11	15	13
NH_3_-N (mg/l)	0.02	0.02	0.07	0.18	0.09	0	0.03	0	0.02	0.02	0.05
TSS (mg/l)	66	55	59	4	42	45	51	50	46	42	46
pH Value	7.40	7.01	6.89	6.86	6.86	6.88	6.77	6.64	6.53	6.45	6.8

### Macroinvertebrate species composition and distribution

**Fig. 2:**
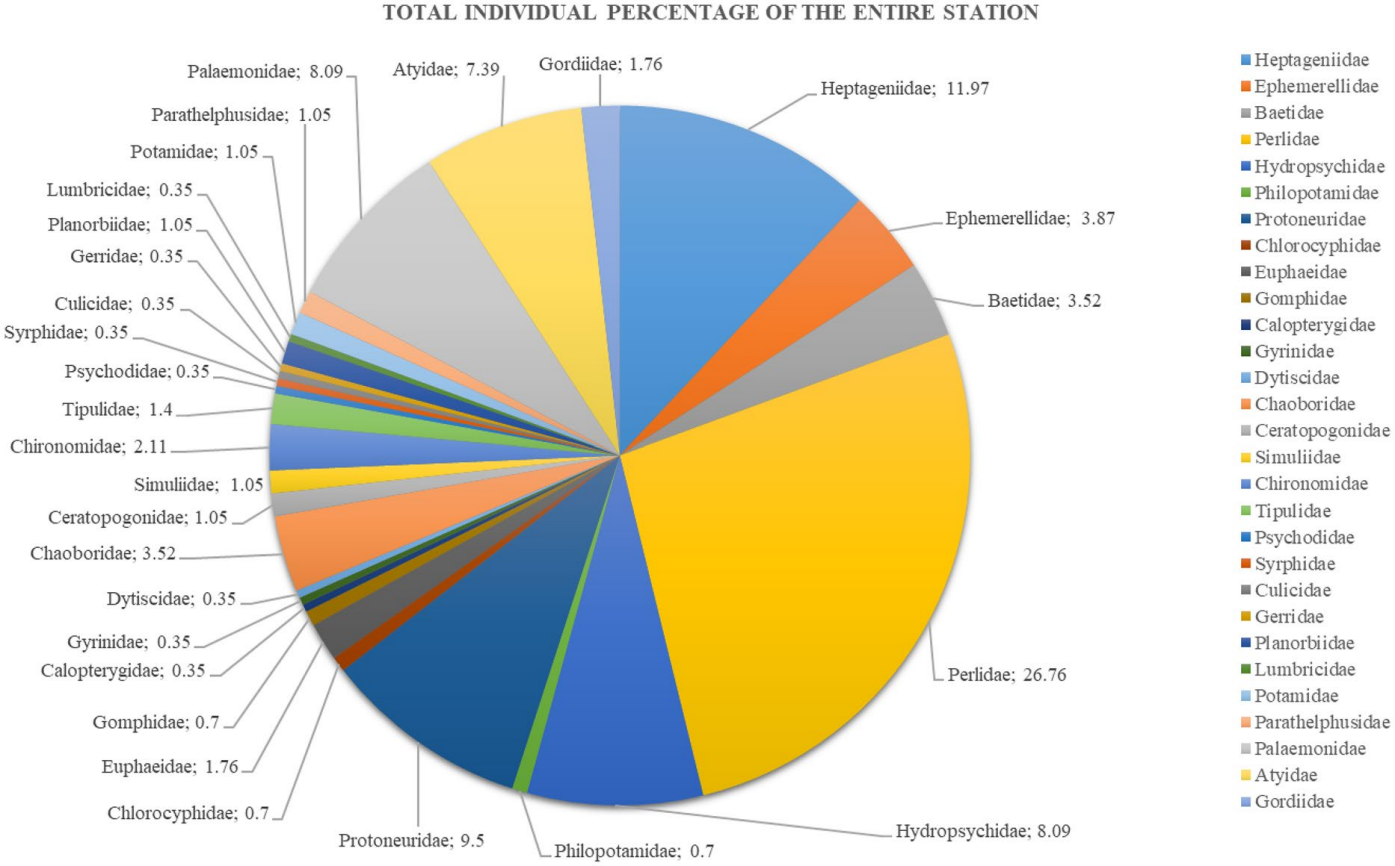
Total individual percentage from all sampling stations

**Fig. 3:**
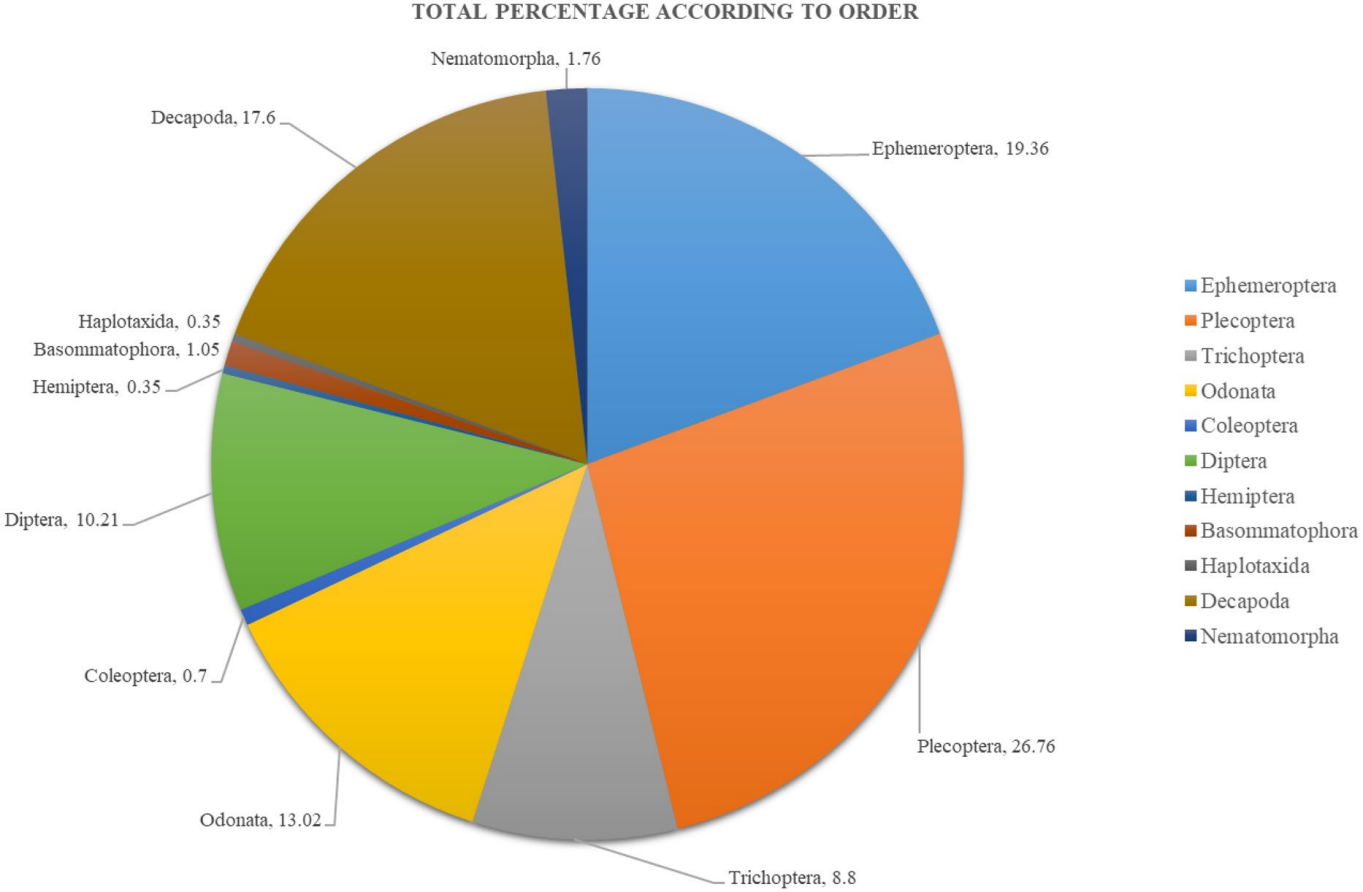
Total percentage according to Order

This study identified a total of 284 macroinvertebrate individuals, which belonged to 11 types of orders and 29 families (Table 3). Figure 2 shows the percentages of macroinvertebrates based on family; meanwhile, Fig. 3 shows the percentages of macroinvertebrates based on order. There were six dominant families based on this study, which were Perlidae with 26.76%, followed by Heptageniidae (11.97%), Protoneuridae (9.50%), Palaemonidae (8.09%), Hydropyscidae (8.09%), and Atyidae with 7.39%. The other families recorded less than 5% of the assemblages. As for the order, there were three orders known to be dominant which were Plecoptera with 26.76%, followed by Ephemeroptera with 19.36%, and Decapoda with 17.60%. The other orders recorded less than 15% of the assemblages.

**Table 3:** Macroinvertebrate species composition and distribution

Order	Family	Sampling station										Total
		1	2	3	4	5	6	7	8	9	10	
Ephemeroptera	Heptageniidae Ephemerellidae Baetidae	2 0 0	5 0 0	1 0 0	10 1 0	0 3 2	2 5 2	7 0 0	0 0 3	3 0 1	4 2 2	34 11 10
Plecoptera	Perlidae	24	11	14	7	5	6	1	3	3	2	76
Trichoptera	Hydropsychidae Philopotamidae	1 0	2 0	6 0	8 2	4 0	0 0	1 0	1 0	0 0	0 0	23 2
Odonata	Protoneuridae Chlorocyphidae Euphaeidae Gomphidae Calopterygidae	3 1 0 0 0	1 0 0 0 0	7 0 2 0 0	3 0 1 0 0	1 1 1 0 0	0 0 0 0 0	2 0 0 0 0	2 0 1 1 1	2 0 0 0 0	6 0 0 1 0	27 2 5 2 1
Coleoptera	Gyrinidae Dytiscidae	0 0	0 0	0 0	0 0	0 0	1 0	0 0	0 0	0 0	0 1	1 1
Diptera	Chaoboridae Ceratopogonidae Simuliidae Chironomidae Tipulidae Psychodidae Syrphidae Culicidae	1 1 1 1 0 0 0 0	0 2 0 1 0 0 0 0	1 0 0 0 0 0 0 0	0 0 1 1 1 0 0 0	1 0 0 2 3 0 0 0	1 0 0 0 0 0 0 0	1 0 0 0 0 1 0 0	3 0 0 0 0 0 0 1	1 0 1 1 0 0 1 0	1 0 0 0 0 0 0 0	10 3 3 6 4 1 1 1
Hemiptera	Gerridae	0	0	0	0	0	0	0	1	0	0	1
Basommatophora	Planorbiidae	0	0	0	0	1	1	1	0	0	0	3
Haplotaxida	Lumbricidae	0	0	0	0	1	0	0	0	0	0	1
Decapoda	Potamidae Parathelphusidae Palaemonidae Atyidae	1 0 3 2	0 0 4 1	0 0 2 2	0 1 6 2	0 0 0 0	0 2 6 4	1 0 2 3	1 0 0 2	0 0 0 3	0 0 0 2	3 3 23 21
Nematomorpha	Gordiidae	3	0	1	1	0	0	0	0	0	0	5
Total		44	27	36	45	25	30	20	20	16	21	284

The composition of macroinvertebrates varied between sampling stations. Based on Fig. 2, the highest numbers of individual macroinvertebrates were found at station no. 4 with 45 individuals, followed by station no. 1 (44), station no. 3 (36), station no. 6 (30), station no. 2 (27), station no. 5 (25), station no. 10 (21), station no. 7 (20), station no. 8 (20), and lastly station no. 9 with 16 individuals. The macroinvertebrate assemblages according to the sampling station are shown in Fig. 3 (a, b, c, d, e, f, g, h, i, and j). The family of Perlidae was dominant at several sampling stations such as station nos. 1, 2, 3, 5, and 6. The highest diversity distribution of macroinvertebrates was at station no. 4 with 14 types of family.

Table 4 shows the values of the Shannon Diversity Index, EPT Index, and EPT percentages from all sampling stations. The value of Shannon Diversity Index showed that a lower index was recorded at upstream river sampling stations such as station no. 1 (1.76), station no. 2 (1.71), and station no. 3 (1.76). On the other hand, the EPT Index showed that most of the downstream sampling stations are zero, except station no. 9 (7). Meanwhile, the EPT percentages showed that upstream sampling stations such as station no. 1 (61.3%), station no. 2 (67%), station no. 3 (58%), station no. 4 (62.2%), and station no. 5 (56%) had higher percentages of EPT compared with the others (ranging between 35 and 50%).

**Table 4:** Value of Shannon Diversity Index, EPT Index and EPT percentages

Index	Sampling station										Average
	1	2	3	4	5	6	7	8	9	10	
Shannon Diversity Index	1.76	1.71	1.76	2.22	2.30	2.10	2.00	2.36	2.10	2.01	2.03
EPT Index	27	18	0	28	7	0	0	0	7	0	8.7
EPT (%)	61.3	67	58	62.2	56	50	45	35	44	48	53

### The relationship between environmental factors and macroinvertebrate communities

**Fig. 4:**
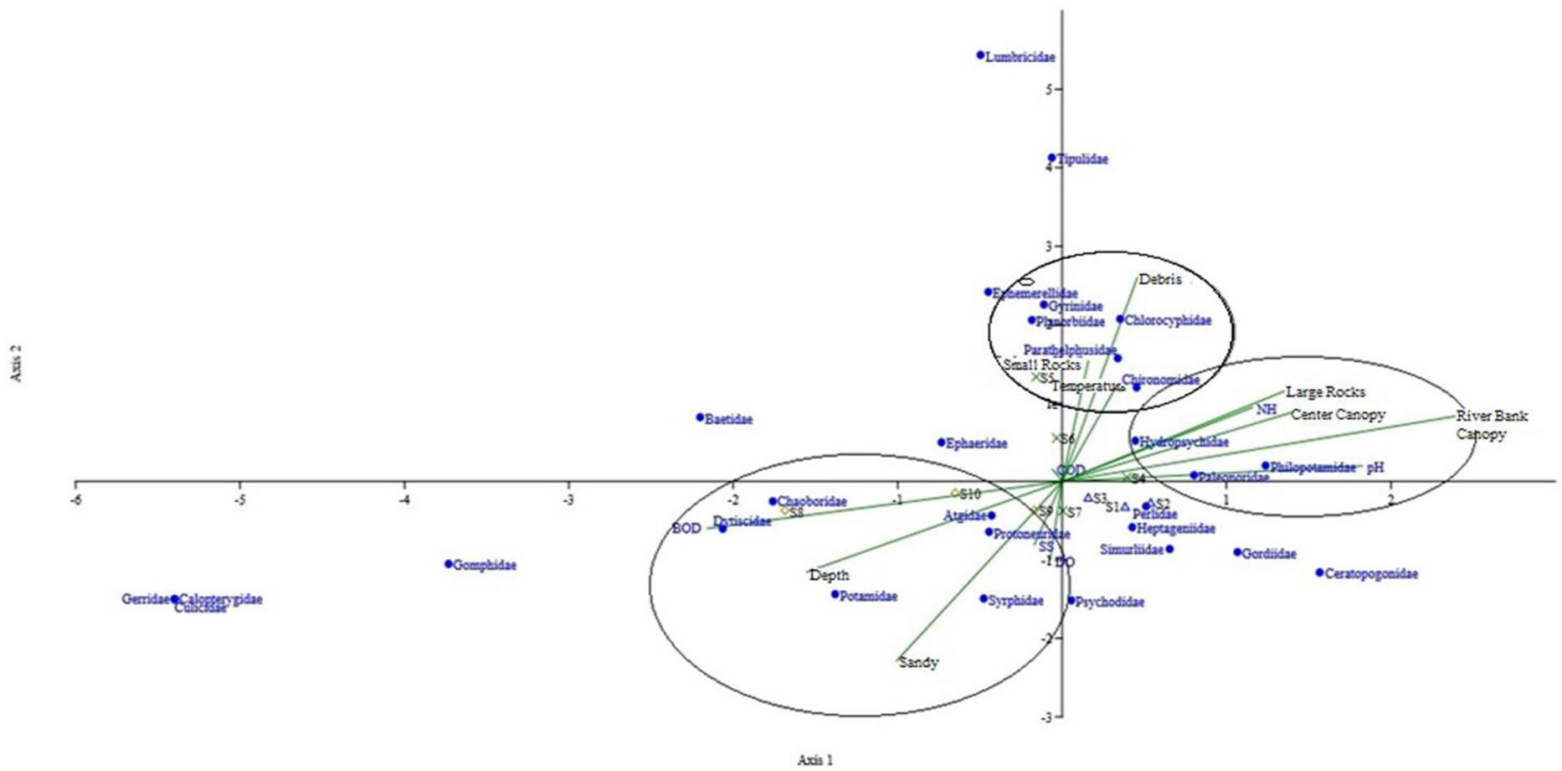
Canonical correspondence analysis of macroinvertebrate assemblages and environmental factors

Based on Fig. 4, it shows that the assemblages of several families of macroinvertebrates such as Dytiscidae, Chaoboridae, Potamidae, Atyidae, Protoneuridae, and Syrphidae were determined by environmental factors such as BOD, river depth, sandy riverbed, TSS, and DO. On the other hand, environmental factors such as small rocks and debris on the riverbed, temperature, and COD clearly determined the assemblage of Chironomidae, Parathelphusidae, Chlorocyphidae, Planorbiidae, Gyrinidae, Ephemerellidae, and Euphaeidae at the Ulu Bendul River. Meanwhile, the families Hydropsychidae, Philopotamidae, and Palaemonidae were determined by the pH, canopy cover, NH_3_-N, and large rocks in the riverbed.

## Discussion

Identifying the determinant environmental factors for the assemblage of the macroinvertebrate community and understanding the relationship between the two are critically significant for environmental monitoring and ecosystem conservation (Wainger et al., 2016). It is important to note that the determinant factors vary based on the water quality and also the habitat characterization (Wang et al., 2003). Different levels of water quality and habitat characteristics will facilitate different types of macroinvertebrate communities (Wang & Tan, 2017). Ulu Bendul River consists of various types of river geomorphological characteristics that literally will introduce different types of species communities, especially for the macroinvertebrates. The morphology and channel characteristics of the Ulu Bendul River are categorized as those of an equatorial tropical river (Vijith & Dodge-Wan, 2018). It has the features of partially eroded mountain belts of Mesozoic and Late Palaeozoic age (Latrubesse et al., 2005). With these kinds of features and characteristics, Ulu Bendul River can be classified as meandering and some segments as tortuously meandering with varied stream valleys. Due to these characteristics and classifications, it is normal to see various types of rock sizes in the riverbed. Plus, the river is surrounded by tropical forest; therefore, the canopy cover at the riverbank of Ulu Bendul River is lush (Othman et al., 2018). On the other hand, according to Tan et al. (2019), the temperature of a tropical river, especially in Malaysia, usually ranges between 21.0 and 24.6 ºC due to the dry–wet conditions of the atmosphere and high precipitation throughout the year.

As with other tropical rivers, Ulu Bendul River has also been affected by human interventions through several activities such as industrialization, commercialization, recreation etc. (Latrubesse et al., 2005). The findings of this study are also in agreement as shown in Table 2, where most of the sampling stations showed a slight degradation of the water quality through six basic parameters, where COD was the main pollutant. Generally, sampling stations that had greater depth had better water quality in terms of DO, COD, and BOD. This was due to the higher volume of water which subsequently increased the DO and decreased COD and BOD. However, the level of COD was found higher compared to BOD, and this shows that the recreational activities alongside the river have produced excessive food waste residue which leads to the increment of COD in the river. TSSs were found to be higher at sampling stations nearer to the upstream end, which may be due to the deforestation activity nearby. Deforestation activity will increase the flow of sediment to the river and will affect the TSS concentration (Yap et al., 2006). On the other hand, the pH value at Ulu Bendul River is less acidic, especially at the sampling stations nos. 3–10. Only the sampling stations no. 1 and no. 2 had standard pH values which are around 7.0–7.5 according to WHO. This situation is due to the increasing human activities such as recreation and fishing closer to the river which leads to the decomposition of organic matter and eventually increases the concentration of carbon dioxide in the river (Ling et al., 2016).

The findings of this study revealed that the diversity distribution and composition of macroinvertebrates varied depending on the habitat characteristics and water quality at the sampling stations. Perlidae, from the order Plecoptera, was the most abundant family in the Ulu Bendul River. According to Ahmad et al. (2015), Plecoptera is one of the most common orders, along with Decapoda, which can be found in Malaysian river water, especially in rivers that flow through the mountain forests. As mentioned above, the composition of macroinvertebrates in the Ulu Bendul River was different at the different sampling stations. The most varied compositions of assemblages of macroinvertebrate communities were found at the sampling station located at the upstream end of the Ulu Bendul River. Plecoptera, Diptera, and Nematomorpha were the orders of macroinvertebrates that were found only upstream of the Ulu Bendul River. According to Hill et al. (2016), these orders dwell in cold river water with less sunlight penetration. Meanwhile, the downstream sampling stations had less varied compositions, and some orders were only found at those stations, such as Coleoptera and Hemiptera. The orders Coleoptera and Hemiptera are commonly found dwelling under rocks at downstream sites in tropical river water (Slimani et al., 2019).

This study showed there was a clear clustering of specific families of macroinvertebrates and the habitat characterization and water quality parameters. Families such as Atyidae, Potamidae, Protoneuridae, Syrphidae, Chaoboridae, and Dytiscidae were grouped with sandy riverbeds, depth of the river, and DO and BOD parameters. The Atyidae (shrimp family), Potamidae (small crabs family), and Protoneuridae (damselflies) were always found dwelling in the river areas with good DO quality. According to Milner et al. (2019), dragonflies need high oxygen concentrations in the water to lay their eggs. Plus, high DO in the water means lower temperatures which are compatible for dragonfly larvae to live and grow. In addition, the existence of the larvae makes the shrimps and small crabs who feed on the larvae able to live abundantly in the same area. The second groups that have been identified were the families of Hydropsychidae, Palaemonidae, and Philopotamidae. Both Hydropsychidae and Philopotamidae are in the order Trichoptera which are known as caddisflies. These two families need a high amount of NH_3_-N in the water for their growth and survival. The existence of Palaemonidae is due to its eating habits; it preys on the smaller macroinvertebrates such as caddisflies (Moorhouse et al., 2014). On the other hand, families such as Chlorocyphidae, Chironomidae, Gyrinidae, Ephemerellidae, and Parathelphusidae are grouped in one cluster along with temperature and small rocks and debris riverbeds. All of the families grouped in this cluster are very dependent on temperature and the type of riverbed in order to lay eggs and for the growth of the larvae. According to Kumar and Khan (2013), temperature is an important factor for most of the families of macroinvertebrates in choosing their egg-laying areas, and a debris-type riverbed provides more food and nutrients needed for the larvae to grow.

## Conclusion

This study confirmed that different families of macroinvertebrates have different determinant factors for assemblages in the recreational river. This study also revealed that the upstream river area gets degraded due to human recreational activities such as camping and fishing. Stream health conditions measured by nutrient concentrations and sediments were clearly influenced by anthropogenic activities in the watershed. This situation has affected the assemblages of macroinvertebrates in the upstream river area. Most of the families of macroinvertebrates are very dependent on environmental factors such as the temperature, DO, NH_3_-N, the type of riverbed, etc. All of these factors are important for the survival of the particular type of macroinvertebrate, plus they are also important for selecting egg-laying areas and for the growth of the larvae.

The findings of this study are extremely important for performing bioassessments and developing indicators for recreational river regions with varying natural settings, as well as the many ways by which anthropogenic activities alter the physicochemical and biological populations of streams. Water quality and biodiversity management and restoration must address land-use processes at larger (e.g., watershed) scales, particularly for streams with low gradients in lowland settings. Minor-scale impacts, such as agricultural operations and tropical forest vegetation along small stream channels with steep slopes in mountain ecoregions, must also be considered in watershed management. This study advises that improved landscape design for watershed management be implemented in order to enhance water quality and physical habitats, and so conserve and restore macroinvertebrate biodiversity.

## Declarations

### Disclaimer

The funders had no role in the design of the study; in the collection, analyses, or interpretation of data; in the writing of the manuscript, or in the decision to publish the results.
